# Effects of *Bacillus velezensis* GUAL210 control on edible rose black spot disease and soil fungal community structure

**DOI:** 10.3389/fmicb.2023.1199024

**Published:** 2023-07-27

**Authors:** Wanpeng Dong, Ting Long, Jinghua Ma, Nan Wu, Weidi Mo, Zhicheng Zhou, Jing Jin, Hongying Zhou, Haixia Ding

**Affiliations:** ^1^Guizhou Botanical Garden, Guiyang, China; ^2^Department of Plant Pathology, College of Agriculture, Guizhou University, Guiyang, China

**Keywords:** soil fungal structure, biological control, *Bacillus velezensis*, rose (*Rosa chinensis*), black spot disease

## Abstract

**Introduction:**

Rose black spot is an economically important disease that significantly decreases flower yield. Fungicide and biological control are effective approaches for controlling rose black spot. The objective of this study was to evaluate the effect of application of biological and chemical control agents, including *Bacillus velezensis* (GUAL210), *Bacillus sp*. (LKW) and fungicide (CP) on the black spot disease and rhizosphere fungal community structure of edible rose.

**Methods:**

In this study, the *R. chinensis* ‘Crimson Glory' was taken as the research object, and the field experiment was designed by randomized block design. The experiment contained 3 treatments (CP, GUAL210, LKW) and 1 control. The control effect and growth promoting effect of fungicide and biological control on rose black spot were compared. The composition and diversity of rhizosphere soil fungal community of different treatments of rose were studied by high-throughput sequencing method. The fungal community composition, correlation of environmental factors and differences in metabolic pathways related to rose disease were analyzed, and the correlation between rhizosphere soil fungal community of rose and biological control of disease was explored.

**Results and discussion:**

Both disease incidence and disease index differed significantly among groups (LKW < GUAL210 < CP < CK), and disease control effect exhibited no significant difference between GUAL210 and LKW (60.96% and 63.86%, respectively). Biological control was superior to chemical control in terms of disease prevention effects and duration, and it significantly increased the number of branches and flowers of rose plants. Ascomycota and Basidiomycota accounted for more than 74% of the total fungal abundance, and the abundance of Ascomycota was highest in CK, followed by GUAL210, CP and LKW, which was consistent with the disease occurrence in each group. The analysis of metabolic pathways showed that the HSERMETANA-PWY in each experimental group was significantly lower than that in control group. The Shannon index in each experimental group was significantly lower than that in control group. PCoA analysis showed that the rhizosphere fungal community structure in each experimental group was significantly different from that in control group. *Trichoderma, Paraphaeosphaeria, Suillus, Umbelopsis* in GUAL210, and *Galerina* in LKW replaced *Mortierella, Pestalotiopsis, Ustilaginoidea, Paraconiothyrium, Fusarium*, and *Alternaria* as dominant flora, and played a nonneglectable role in reducing disease occurrence. The difference in rhizosphere fungal community structure had an important impact on the incidence of rose black spot disease. Biological control is crucial for establishing environment-friendly ecological agriculture. GUAL210 has promising prospects for application and development, and may be a good substitute for chemical control agents.

## 1. Introduction

Edible roses generally refer to *Rosa rugosa, Rosa chinensis, Rosa* sp., and their hybrids belonging to *Rosa L*, Rosaceae, which are widely used in the food, medicine, cosmetics, and healthcare industry. *R. chinensis* “Crimson Glory” is generally used to produce dry flower tea and candied flower and has antioxidant, anti-inflammatory, and other healthcare effects (Dong et al., 2020). Fungal diseases strongly affect the growth of “Crimson Glory” during field planting (Liu et al., 2019), among which black spot disease is most harmful to rose plants. It mainly infects leaves and branches, causing severe defoliation or even death of the plants. Studies have shown that black spot disease is mainly spread by rain splash, irrigation water, pruning tools, and diseased residues. Rainy and humid conditions are conducive to the occurrence of this disease. The pathogen generally overwinters in the form of sclerotia, mycelia, and conidia in plant-diseased tissues in the soil (Zhang et al., 2019). In the previous study, *Marssonina rosae* has been identified as the pathogen responsible for rose black spot disease through isolation and purification, pathogenicity determination, morphological identification, and multi-gene sequence analysis (Mo et al., 2022). Moreover, a study has shown that the pathogenesis of rose black spot is related to soil fungal community composition (Liu and Zhang, 2020).

The plant rhizosphere is a complex microecosystem whose dynamic balance is maintained by the interactions between pathogens, probiotics, and host plants. Rhizosphere microbial ecosystems are closely related to plant nutrition and health. The interactions between rhizosphere fungi can be classified as antagonism and synergy (Bais et al., 2006). In the rhizosphere microdomain, it is difficult for an antagonistic bacterial population to proliferate arbitrarily due to the interactions among diverse microbial groups. Studies have found that a single antagonistic bacterial population usually needs to reach a certain abundance to effectively resist pathogenic infection (Berendsen et al., 2012). The interactions among different bacterial populations contribute to the production of antifungal substances, thereby enhancing the inhibitory effect on pathogenic fungi (Boer et al., 2007). Rhizosphere microorganisms play a regulatory role in mycorrhizal synthesis, and some bacterial groups, such as *Bacillus*, play an important role in promoting mycorrhizal synthesis and plant growth (Frey-Klett et al., 2007). Plant growth-promoting rhizobacteria (PGPR), such as *Bacillus amyloliquefaciens*, can promote plant growth, increase crop yield, and reduce disease occurrence by producing indole acetic acid (IAA) and metabolites with anti-pathogenic fungi activity (Wang et al., 2019). Luo et al. (2011) has isolated a strain of PGPR *Bacillus subtilis* from the soil, which can produce the lipopeptide compound Bacillomycin L and inhibit pathogenic fungi. The growth of pathogens can induce plant disease resistance. *Bacillus subtilis* secretes antifungal substances, such as subtilisin and polymyxin, when colonizing the rhizosphere of plants, induces plant defense systems to resist pathogenic invasion, and forms a competitive relationship with pathogens (Yang, 2015). To date, few studies have focused on the effect of biocontrol bacteria on rose fungal diseases and the interactions between biocontrol bacteria and pathogenic fungi. Our previous experimental results have also shown that *B. amyloliquefaciens* has a significant control effect on rose black spot (Mo et al., 2022). *B. velezensis* is considered to be a novel biocontrol *Bacillus* with 99% similarity to *B. amyloliquefaciens* (Gao, 2019). *B. velezensis* GUAL210 can occupy favorable ecological positions, such as stomatal positions, and reduce the abundance of other flora, thus having a biological control effect on pathogens. However, the effect of *B. velezensis* GUAL210 on plant rhizosphere fungi has rarely been studied.

Soil fungal diversity and community structure play an important role in crop production and ecosystem stability (Wang and Bau, 2014). In addition, fungicide treatment is considered to be one of the main abiotic factors, affecting the structural and functional diversity of rhizosphere microbial communities (Berg and Smalla, 2009). To date, fungicides have been widely used to control fungal diseases of roses due to their advantages of quick results, convenient use, and low cost. However, long-term use of fungicides may lead to drug resistance (Zhang et al., 2019). Therefore, biological control, which is harmless, environment-friendly, and long-lasting, has received increasing attention recently. To date, only a few studies have explored the relationship between biological control against rose diseases and rhizosphere fungal communities. Thus, it is crucial to study the effect of chemical fungicide and biological control on rose black spot and rhizosphere fungal communities. By comparing the contribution of chemical fungicides and biological control to plant disease prevention and growth promotion, this study discussed the relationship between fungal communities and the disease resistance of roses. Hopefully, this study will provide a new strategy for plant disease management.

## 2. Materials and methods

### 2.1. Experimental site

The experiment was carried out in Haohuahong village (26°11′N, 106°40′E, altitude 957 m), Huishui County, Guizhou Province, China from July 2019 to September 2021. The study area has a subtropical monsoon humid climate. The soil at the test site is yellow loam with uniform fertility, in which the average organic matter content is 92.7 g/kg, the average pH is 6.4, the average alkaline nitrogen is 116.2 mg/kg, the average available phosphorus is 89.7 mg/kg, and the average available potassium is 237.6 mg/kg.

### 2.2. Material and management

#### 2.2.1. Material

The experimental plant was *R. chinensis* “Crimson Glory”, which was grafted with the same rootstock. A total of 1,200 *R. chinensis* plants with a plant height of >27 cm and a stem diameter of >0.6 cm were planted at 1 × 2.5 m spacing per mu at the test site.

#### 2.2.2. Strain and growth conditions

*B. velezensis* GUAL210 was isolated from the rhizosphere of healthy pepper plants using the dilution-plate method (Zhou et al., 2023). It was cultivated overnight in a shaker at 37°C and 200 r·min^−1^ in LB medium solidified with 1.5% agar for routine growth and preserved in 15% glycerol at −80°C (Huang et al., 2022).

#### 2.2.3. Experimental design and methods

**Figure 1:**
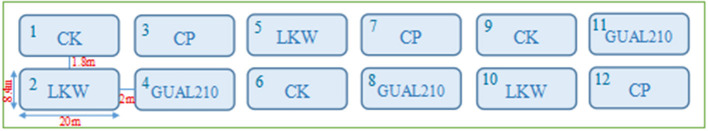
The design diagram of the experimental field.

The experiment contained three treatments and one control as follows: (1) applications of a chemical fungicide, recorded as CP; (2) applications of biocontrol bacteria “*B. velezensis* GUAL210” obtained from College of Agriculture, Guizhou University, recorded as GUAL210; (3) applications of a commercially available biological control agent “Lvkangwei” whose active ingredient is *Bacillus sp*., recorded as LKW; and (4) a blank treatment, recorded as CK (Table 1). Each treatment and control were performed in three replications (Figure 1). The number of plants in each plot was investigated (>200 plants). The first root irrigation and spraying were carried out in June 2021 during the early stage of the rose black spot when the leaves displayed no symptoms. The first spraying was performed after uniform pruning and then was conducted every 20 days.

**Table 1:** Processing methods of each group.

**Block**	**Group**	**Treatment method**	**The number of treatments applied**
3/7/12	CP	Myclobutanil 2% Triadimefon 10% (5,000 times diluted) and Mancozeb 80% (2,000 times diluted)	After pruning, repeated every 20 days for 3 times.
4/8/11	GUAL210	*B. velezensis* GUAL210 (15-fold dilution, 1.33 ^*^ 10^7^ CFU/mL)	Ditto
2/5/10	LKW	*B. sp*. (Effective bacteria number 10 billion/g, 75 times dilution)	Ditto
1/6/9	CK	–	–

Compound fertilizer (N:P:K = 15:15:15) was added during pruning, and water-soluble foliar compound fertilizer (trace elements, alginate, small molecular organic carbon, hydrolyzed organic matter, and free amino acid) was added after spraying. Other field management measures were carried out by local production programs.

#### 2.2.4. Investigated indexes

The condition of experimental plants was investigated after 60 and 105 days of the final spraying, respectively. The total number of plants, diseased plants, and disease grades were recorded. Specifically, disease grade was determined according to the “disease severity grading standard” (Table 2) (Yang et al., 2020). In each plot, growth traits, such as plant height, diameter, and branch number, were measured based on 15 randomly selected plants. Disease incidence, disease index, and relative control effect were calculated as follows:

**Table 2:** Classification standard of disease severity.

**Grade of disease**	**Occurring degree**
Level 0	The whole plant was disease-free
Level 1	The diseased leaves account for < 1/10 of the total leaves.
Level 2	The diseased leaves account between 1/10 and 1/4 of the total leaves.
Level 3	The diseased leaves account between 1/4 and 1/2 of the total leaves.
Level 4	The diseased leaves account between 1/2 and 3/4 of the total leaves.
Level 5	The diseased leaves account for more than 3/4 of the total leaves. The diseased leaves fell off seriously and the plant died.

**Incidence** = (number of diseased plants/total number of investigated plants) × 100%

**DI** (Disease index) = ∑ (number of diseased plants or leaves at all levels × disease level)/(total number of investigated plants or leaves × highest level) × 100

**RCE** (Relative Control Effect) = (Disease Index of Control Area – Disease Index of Treatment Area)/Disease Index of Control Area × 100%

### 2.3. Sample collection and processing

Within 1 month after the investigation, a five-point sampling method was used to collect the rhizosphere soil of roses in each plot, and the whole roots of roses were dug out with a sampling shovel. The loose soil on the root surface was removed, and the soil sticking to the roots was collected. The samples were passed through a 20-mesh sieve to remove impurities. In total, 10 g samples were collected for each group, placed in a 15 ml round-bottom centrifuge tube, sealed with a sealing film, and transported to the laboratory of Wekemo Tech Group Co., Ltd., Shenzhen, China, with dry ice for DNA extraction and quality inspection. Replicated samples were stored in a refrigerator at −80°C for later use.

Soil samples were taken from each plot at a depth of 10–20 cm, according to the S-shaped distribution method. After the debris was removed, they were evenly mixed and put into a sterilized sampling bag. Soil organic matter content was determined by the potassium dichromate volumetric method, and the soil pH was determined by the conventional method (Qiao, 2012).

### 2.4. Nucleic acid extraction and sequencing

Total DNA was extracted according to the instructions of the OMEGA Soil DNA Kit (D5625-01) (Omega Bio-Tek, Norcross, GA, USA), checked on a NanoDrop D-2000 spectrophotometer (NanoDrop Technologies, Wilmington, DE) using agarose gel electrophoresis and uniformly diluted to 20 ng/μl. The internal transcribed spacer (ITS) sequences (primers: GGAAGTAAAAGTCGTAACAAGG/GCTGCGTTCTTCATCGA TGC) of fungi were amplified by PCR.

### 2.5. Data analysis

ANOVA analysis, multiple comparisons, and SigmaPlot drawing were applied to analyze the data on growth traits, including disease incidence, disease index, and relative control effect in Microsoft Excel and SPSS. According to the index and barcode information, the original sequence was preliminarily screened. DADA2 from QIIME2 (https://view.qiime2.org) was used to perform primer removal, quality filtering, denoising, splicing, and chimera and singleton removal, and the results were clustered to obtain amplicon sequence variants (ASVs) at 97% similarity level (Callahan et al., 2016).

The QIIME2.0 software package was used to perform OTU clustering on the sequenced fragments. The representative sequences of OTU were compared with the GreenGenes database to obtain the corresponding taxonomic units of OTU and their corresponding abundance information. The observed OTUs and Shannon and Simpson indexes were calculated to evaluate the diversity of fungal flora in the samples. PCoA analysis, ANOVA, Kruskal–Wallis, and LEfSe tests were used to identify fungi with different abundances between groups and samples. A correlation heatmap was used to analyze the structure of the associated flora and other key factors and find the flora with a positive or negative correlation with the external factors of the sample. We extended the analysis to predict functional genes using Phylogenetic Investigation of Communities by Reconstruction of Unobserved States (PICRUSt2). The data were analyzed on the free online platform of Wekemo Bioincloud.

## 3. Results

### 3.1. Effects of chemical and biological control on rose black spot disease

According to the survey, cases with 4–5 disease grades and a high incidence of rose black spot disease accounted for the highest proportion in CK. The disease incidence was highest in CK, followed by CP, GUAL210, and LKW. The DI in CK, CP, LKW, and GUAL210 reached 12.02, 8.09, 5.36, and 5.79 after 60 days of the final spraying and reached 14.83, 9.13, 5.61, and 6.03 after 105 days of the final spraying, respectively. The subsequent disease incidence and DI under the treatment of chemical control were significantly higher than those under the treatment of biological control. The RCE in GUAL210 and LKW were 60.96 and 63.86%, respectively (Table 3). The subsequent RCE was relatively stable and exhibited no significant difference between the two groups. The RCE under the treatment of chemical control was 45.44% and decreased to 38.42% after 105 days. Biological control was significantly superior to chemical control in terms of disease prevention effect and duration.

**Table 3:** Rose black spot disease and control effects among groups.

**Treatment**	**Incidence (%)**		**DI**		**RCE (%)**	
	**60 days**	**105 days**	**60 days**	**105 days**	**60 days**	**105 days**
CP	78.34 ± 1.67b	84.23 ± 1.53b	8.09 ± 0.24b	9.13 ± 0.42b	45.44 ± 4.13b	38.42 ± 2.37b
GUAL210	66.72 ± 1.16c	67.64 ± 1.79c	5.79 ± 0.17c	6.03 ± 0.21c	60.96 ± 3.37a	59.34 ± 1.87a
LKW	49.34 ± 0.93d	51.75 ± 1.34d	5.36 ± 0.15c	5.61 ± 0.19c	63.86 ± 2.36a	62.17 ± 2.66a
CK	98.54 ± 1.30a	99.28 ± 1.97a	12.02 ± 0.77a	14.83 ± 0.79a	–	–

If there are the same letters above the two groups, the difference is not significant, otherwise the difference is significant.

### 3.2. Effect of chemical and biological control on promoting rose growth

**Figure 2:**
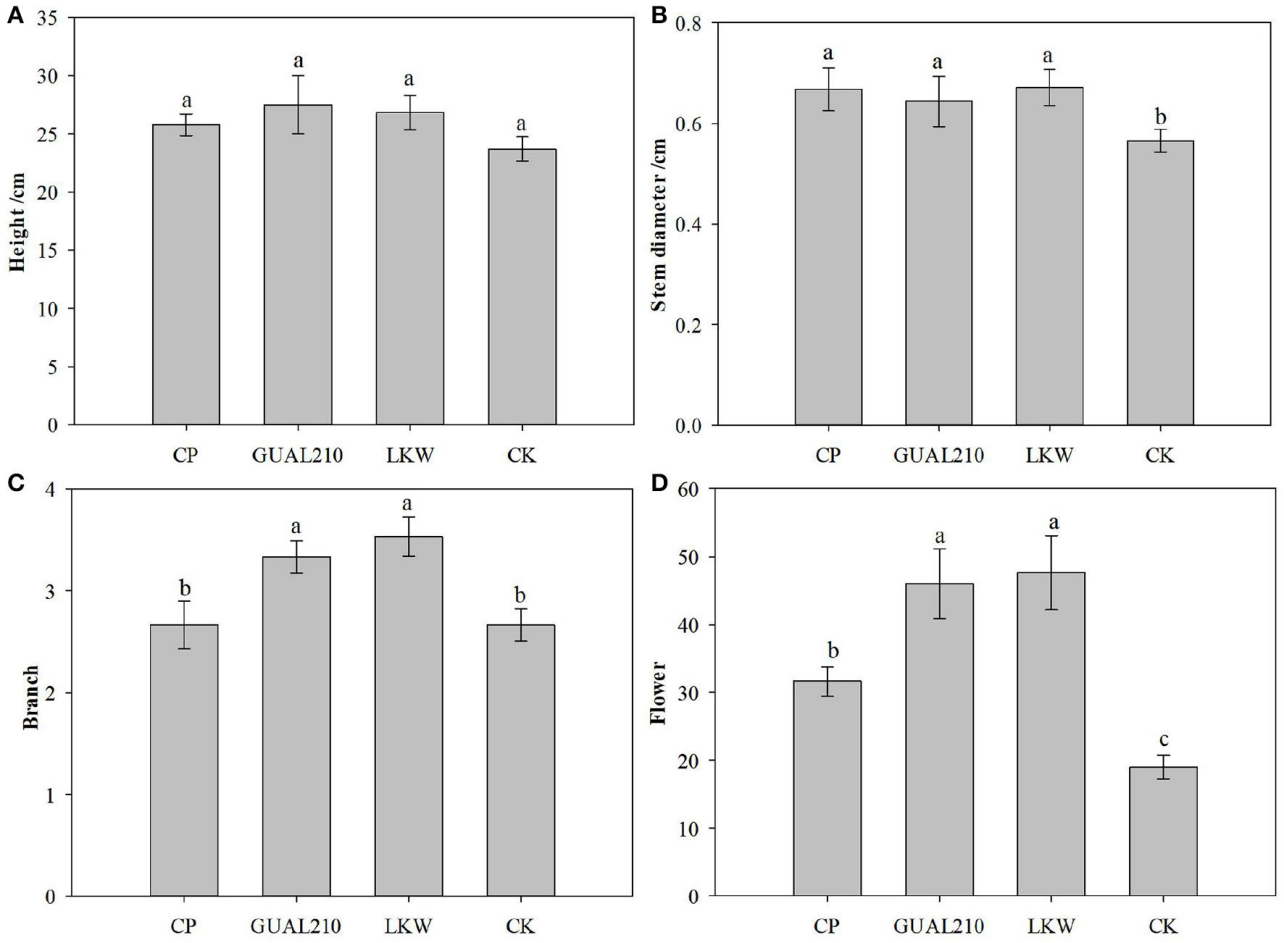
Effects of each treatment on the growth and flower number of roses. If there are the same letters above the two groups, the difference is not significant, otherwise the difference is significant, same as Figure 11. **(A)** Effects of each treatment on the plant height of roses. **(B)** Effects of each treatment on the stem diameter of roses. **(C)** Effects of each treatment on the number of branches of roses. **(D)** Effects of each treatment on the number of flowers of roses.

There was no significant difference in plant height among groups (Figure 2). There was a significant difference in stem diameter between the experimental group and the control group (*p* < 0.05). There was a significant difference in the number of branches between “GUAL210, LKW” and “CP, CK” (*p* < 0.05). There was a significant difference in flower volume between “GUAL210, LKW” and “CP, CK” (*p* < 0.05). These results showed that each treatment had a non-neglectable effect on the growth of roses, especially on the diameter index. Biological control had a significant effect on the number of branches and flowers of roses. Overall, compared with chemical control, biological control had a more favorable effect on the growth and flowering of roses.

### 3.3. Analysis of species composition and fungal community structure

#### 3.3.1. Amplification, sequencing, and annotation

Clear bands of 280 bp were obtained by PCR amplification for fungi. 1,276,950 ITS sequence matching primers were obtained by sequencing (average 70,942 per sample, ranging from 61,491 to 85,635), and 727,826 OTUs were generated by removing low-quality sequences and chimeras (average 40,435 per sample).

#### 3.3.2. Analysis of common species in each group

**Figure 3:**
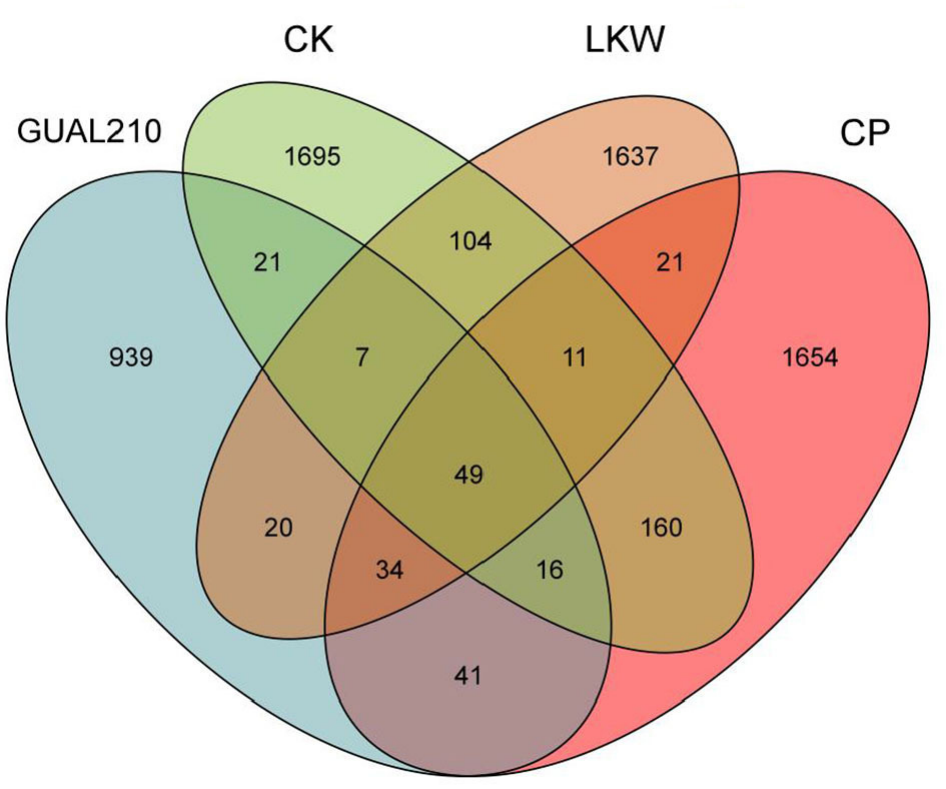
Venn diagram of OTUs.

The total number of OTUs was highest in CK, followed by CP, LKW and GUAL210 (Figure 3). The number of common fungal OTUs was 49, and that of fungal OTUs specific to GUAL210, LKW, CP, and CK was 939, 1,637, 1,654, and 1,695, respectively, indicating that the number of OTU species of rhizosphere fungi was quite different among the groups. The number of OTU species of rhizosphere fungi was the lowest in GUAL210, implying that the application of GUAL210 reduced the abundance of fungi.

#### 3.3.3. Species distribution at the phylum level in each group

**Figure 4:**
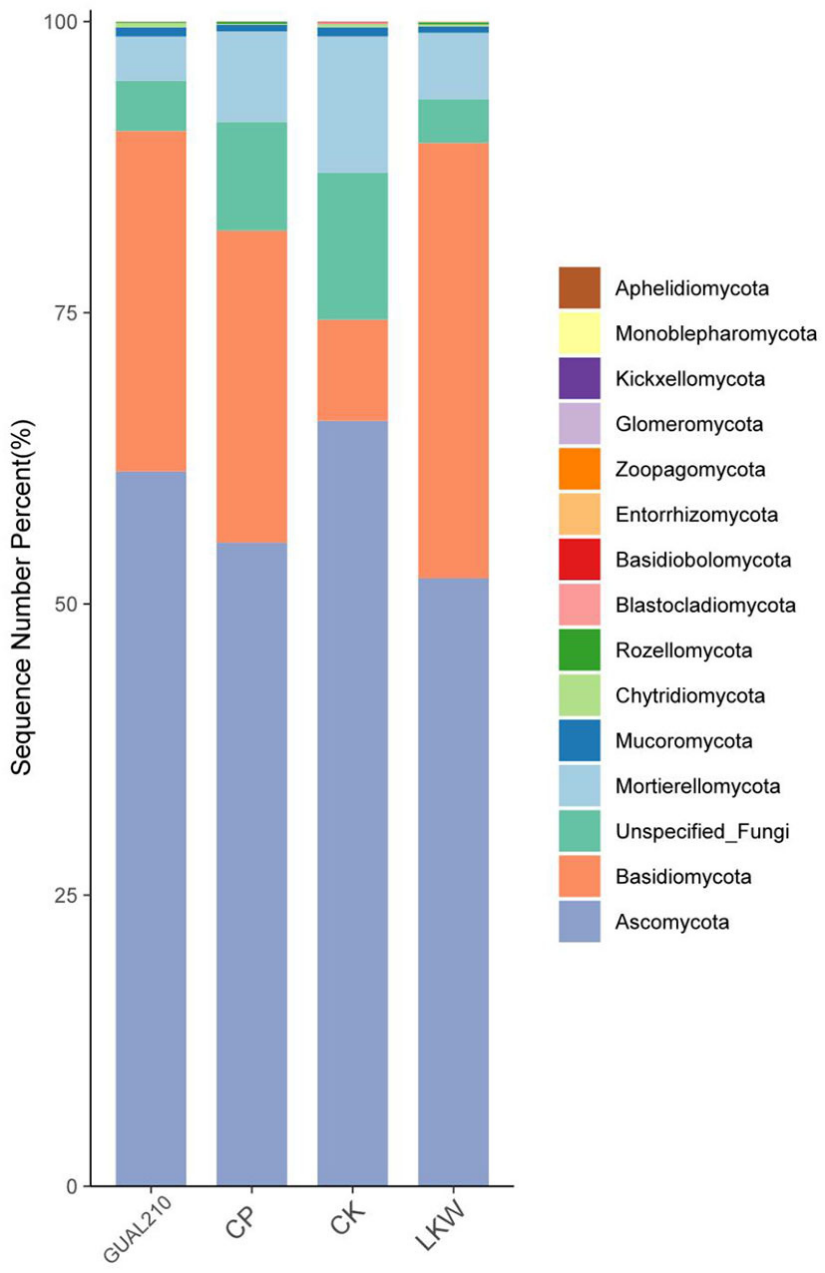
The relative abundance of the top 20 ASV species at the phylum taxonomic level.

The relative abundance of the top 20 ASV (Amplicon Sequence Variant) species was calculated at the phylum level (Figure 4). The dominant fungal flora in each group was Ascomycota, Basidiomycota, Mortierellomycota, Mucoromycota, Chytridiomycota, and Rozellomycota, among which Ascomycota and Basidiomycota accounted for more than 74% of the total fungal abundance. The relative abundance of Basidiomycota in LKW was non-significantly higher than that in other experimental groups and was significantly higher than that in the control group. Most pathogens, such as *M. rosae*, belonged to the phylum Ascomycota. The abundance of Ascomycota was the highest in CK, followed by GUAL210, CP, and LKW.

#### 3.3.4. Characteristic fungi at the genus level in each group

**Figure 5:**
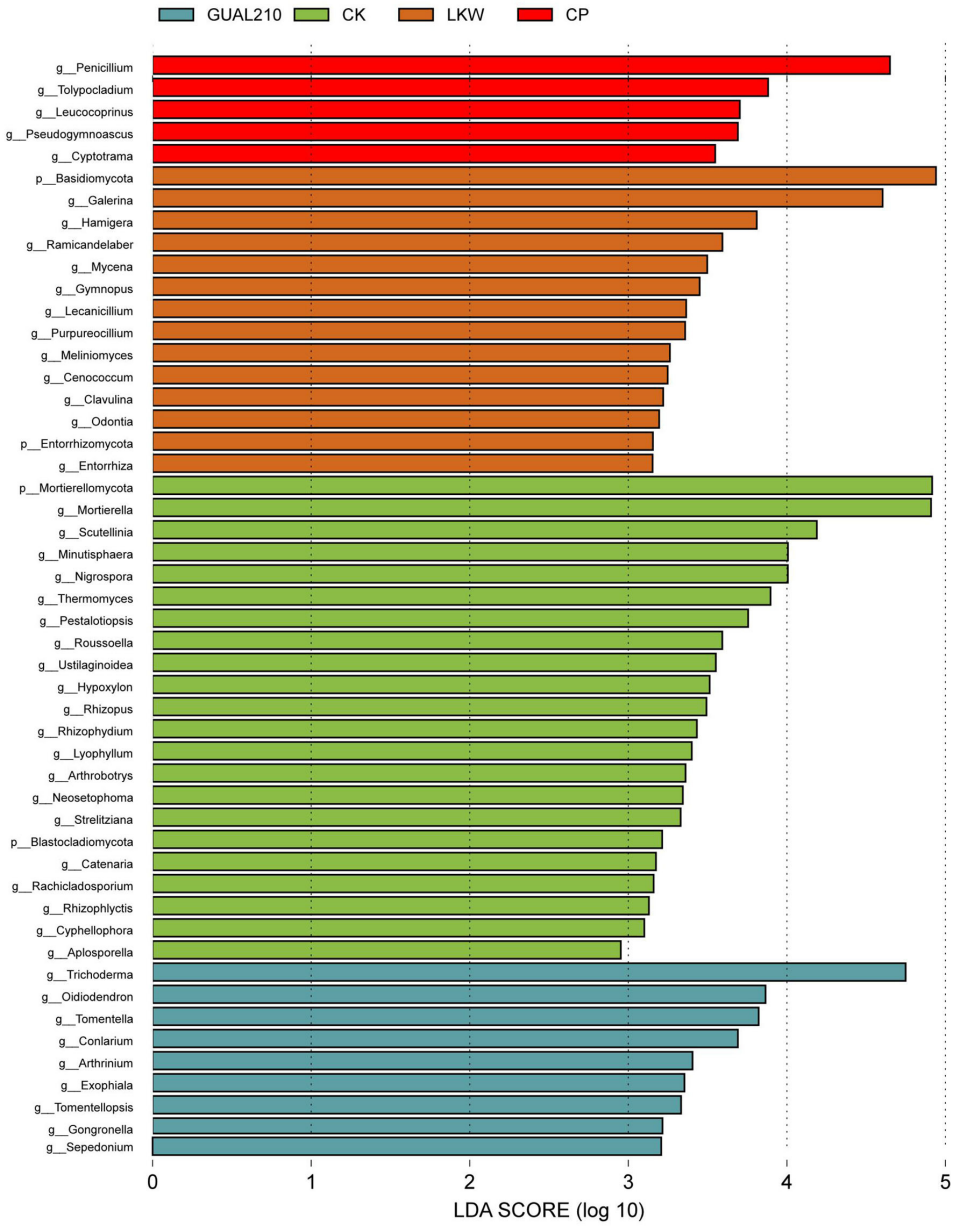
LEfSe analysis of the Cladogram. Each transverse column represents a species, and the length of the column corresponds to the LDA value. The higher the LDA value, the greater the difference. The color of the column corresponds to the group of characteristic microorganisms to which the species belongs, and the characteristic microorganisms (biomarkers) indicate species with a relatively high abundance in the corresponding group.

The results of LEfSe analysis showed that at the genus level (Figure 5), *Trichoderma* had the highest relative abundance among characteristic microorganisms in GUAL210 (LDA SCORE > 4), followed by *Oidiodendron, Tomentella, Conlarium, Arthrinium, Exophiala, Tomentellopsis, Gongronella*, and *Sepedonium. Galerina* had the highest relative abundance in LKW (LDA SCORE > 4), followed by *Hamigera, Ramicandelaber, Mycena, Gymnopus, Lecanicillium, Purpureocillium, Meliniomyces, Cenococcum, Clavulina, Odontia*, and *Entorrhiza*. *Penicillium* had the highest relative abundance in CP (LDA SCORE > 4), followed by *Tolypocladium, Leucococoprinus, Pseudogymnoascus, Cyptotrama*, and *Paraconiothyrium*. *Mortierella* and *Scutellinia* had the highest relative abundance in CK (LDA SCORE > 4), followed by *Thermomyces* and *Pestalotiopsis*.

### 3.4. Analysis of biodiversity and species differences

**Figure 6:**
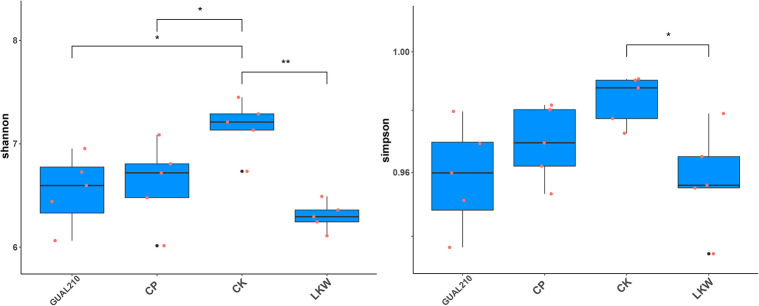
Multiple comparisons of the Shannon and Simpson indexes between groups. *, ** means *p* < 0.05, *p* < 0.01, the same below.

The diversity of the fungal community in each group is shown in Figure 6. Shannon index was the highest in CK, followed by CP, GUAL210, and LKW. Specifically, CP and GUAL210 had a significantly lower Shannon index than CK (*p* < 0.05), LKW had a significantly lower Shannon index than CK (*p* < 0.01), and GUAL210 and LKW exhibited no significant difference in Shannon index. The trend of the Simpson index was almost similar to that of the Shannon index in each group. The results showed that both chemical and biological control agents reduced the rhizosphere fungal diversity of rose plants.

**Figure 7:**
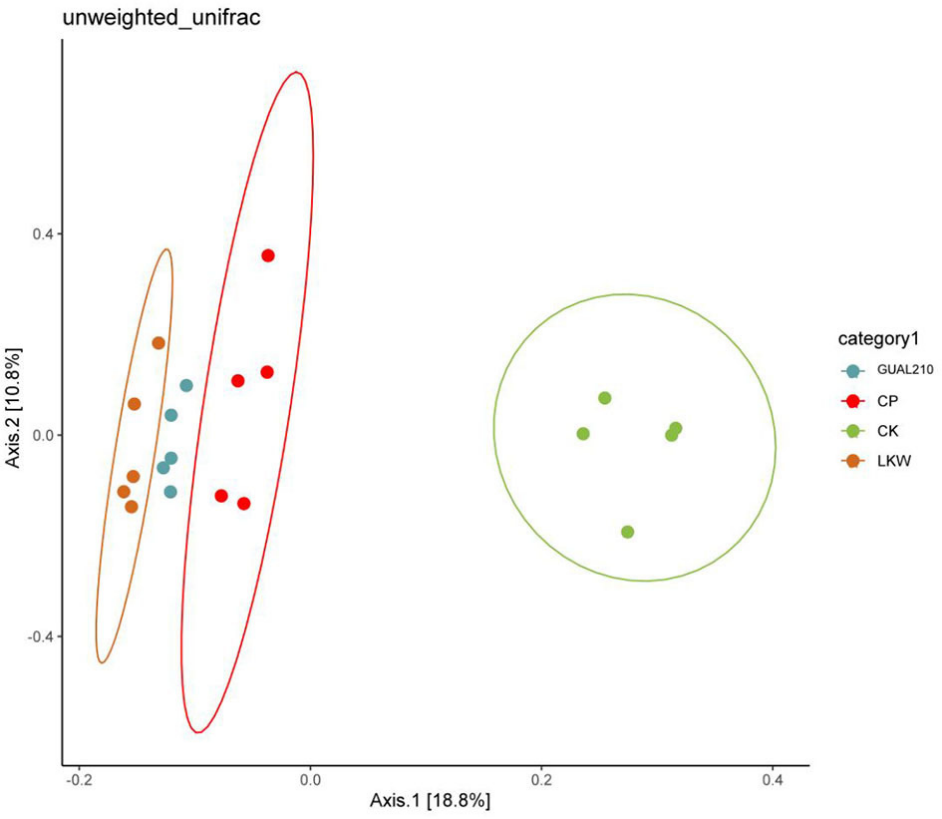
2D_PCoA analysis between the groups.

Based on the distance matrix and principal coordinate analysis (PCoA), the diversity differences between different samples were analyzed. Fungi contained 30% of the sample variation data that could be explained (Figure 7). The distance between GUAL210 and LKW was close, and CP was slightly farther from LKW, and all three groups deviated from the CK. The results showed that there was a large difference in species composition between each experimental group and control group.

### 3.5. Phylogenetic analysis of specific species

**Figure 8:**
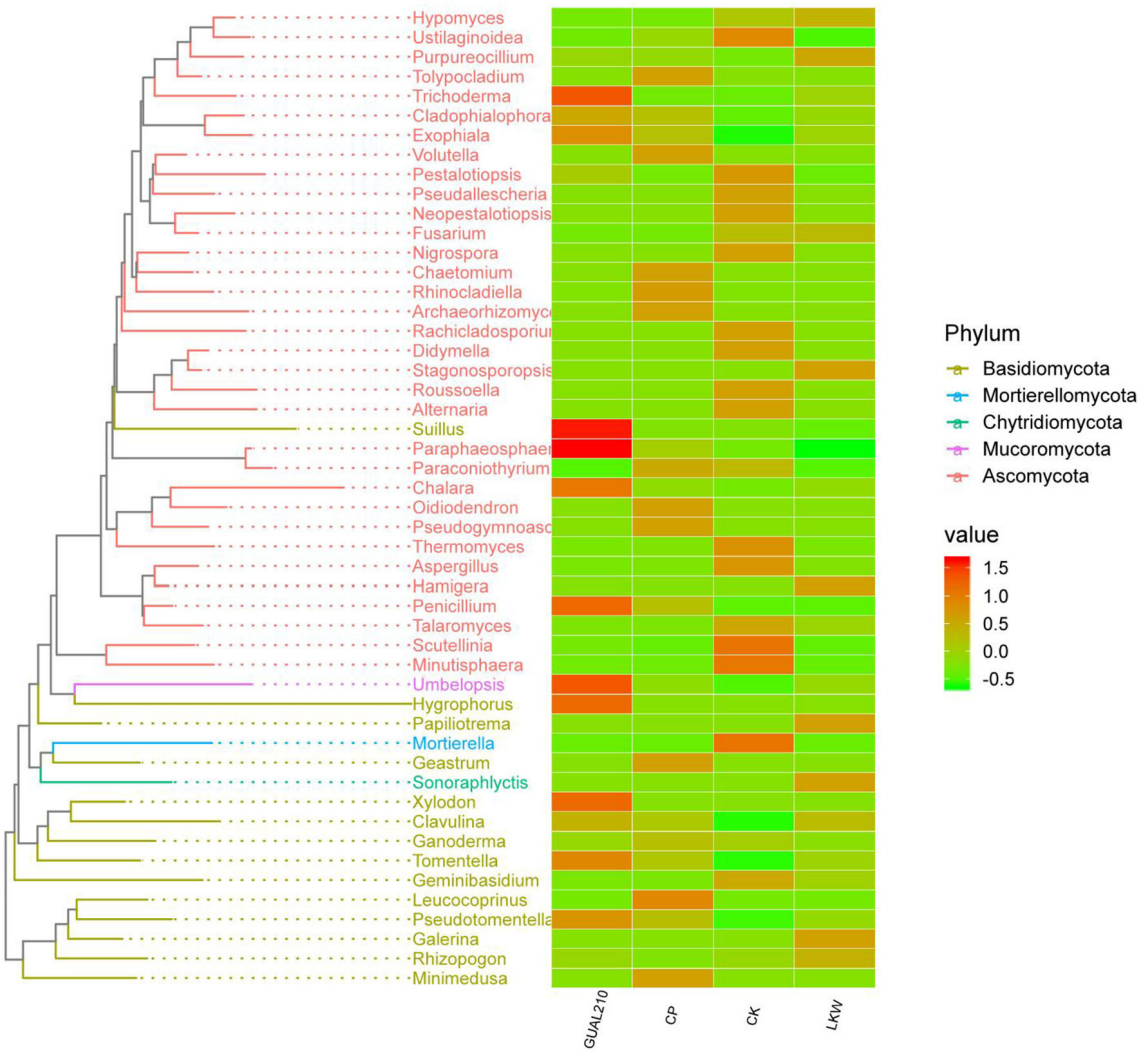
Phylogenetic tree and intergroup abundance distribution heat map. The drawing of the evolutionary tree is carried out by the R language GGTree package. Representative sequences of OTUs of interest are selected for phylogenetic analysis (the OTU with the highest abundance is selected for each genus as the representative OTU, and then the top 50 genera with the highest abundance are selected), to draw the evolutionary relationship tree. At the same time, the absolute abundance of OTUs in each group is displayed with a heat map.

The phylogenetic analysis showed that the abundance of *Trichoderma* in GUAL210 was significantly higher than that in CP and CK. The highest abundance of *Suillus, Paraphaeosphaeria, Chalara, Penicillium, Umbelopsis, Hygrophorus, Xylodon, Tomentella*, and *Exophiala* was observed in GUAL210, while the lowest abundance of *Fusarium* was observed in GUAL210. The abundance of *Purpureocillium, Stagonosporopsis, Hamigera, Papiliotrema, Sonoraphlyctis*, and *Galerina* was the highest in LKW. The abundance of *Mortierella, Minutisphaera, Scutellinia, Ustilaginoidea, Thermomyces, Pestalotiopsis*, and *Aspergillus* was the highest in CK. The abundance of *Leucococoprinus, Minimedusa, Geastrum, Tolypocladium*, and *Rhinocladiella* was the highest in CP (Figure 8).

### 3.6. Analysis of correlation between indicators

**Figure 9:**
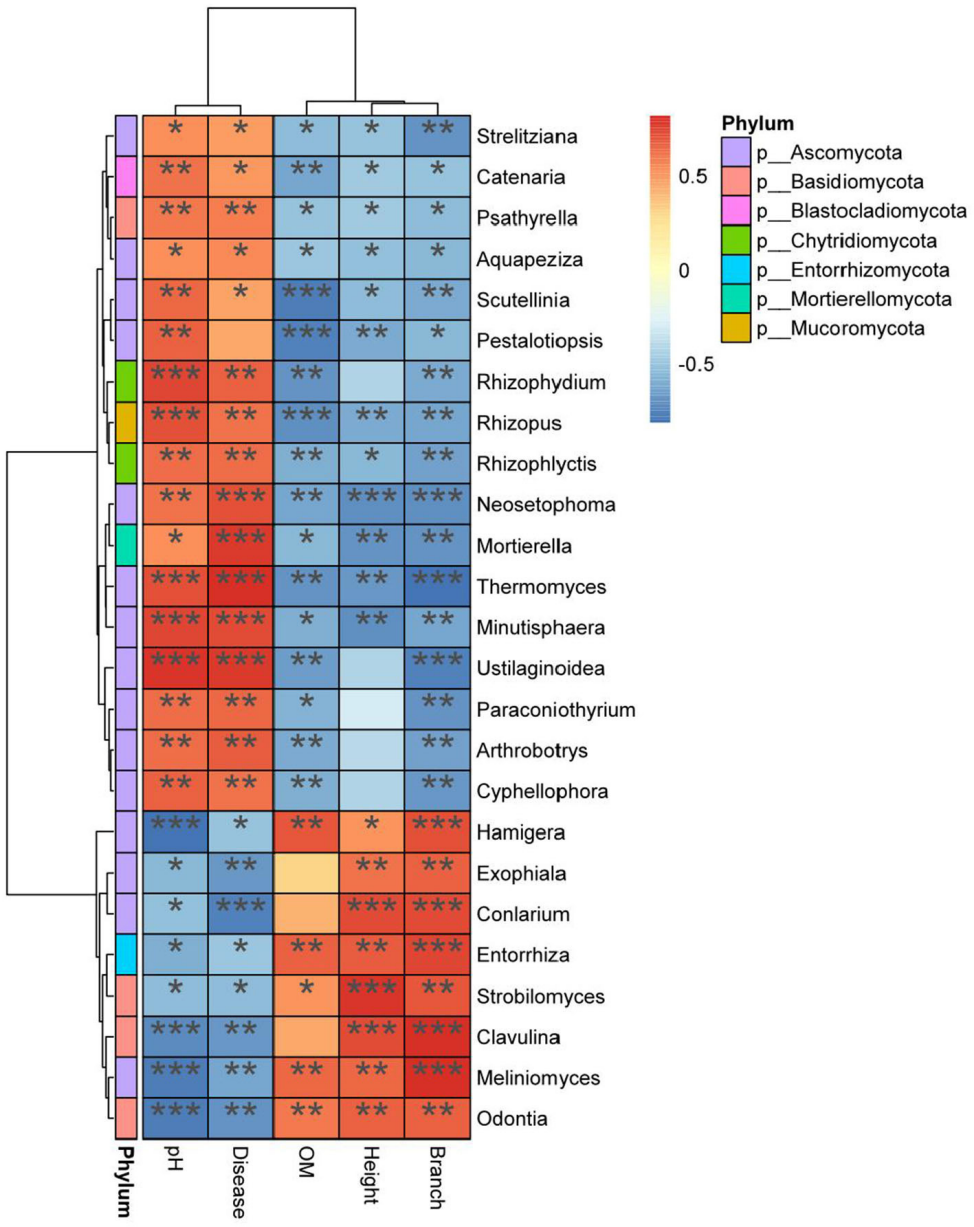
Heat map analysis of correlation among indexes. *X* axis for environmental factors, *Y* axis for species. *R* value (rank correlation) and *P* value are obtained by calculation. The *R* value is displayed in different colors in the figure. ^*^, ^**^, and ^***^ means *p* < 0.05, *p* < 0.01, and *p* < 0.001 respectively. The right legend is the color interval of different *R* values. At the same time, the color bar on the left marks the classification of the species.

*Hamigera, Exophiala, Entorrhiza, Strobilomyces, Conlarium, Clavulina, Meliniomyces*, and *Odontia* were significantly positively correlated with plant height, branch number, and other growth traits (*P* < 0.05) and significantly negatively correlated with DI (*P* < 0.05) (Figure 9). *Hamigera, Entorrhiza, Meliniomyces*, and *Odontia* were significantly positively correlated with soil OM content (*P* < 0.05). *Minutisphaera, Thermomyces, Mortierella*, and *Neosetophoma* were significantly negatively correlated with plant height and branch number (*P* < 0.05). The analysis of indicators showed that *Mortierella, Chaetomiaceae*, and *Pyronemataceae* had the highest correlation with pH and DI, and all of them were significantly positively correlated with DI (*P* < 0.05).

### 3.7. Analysis of functional differences between groups

**Figure 10:**
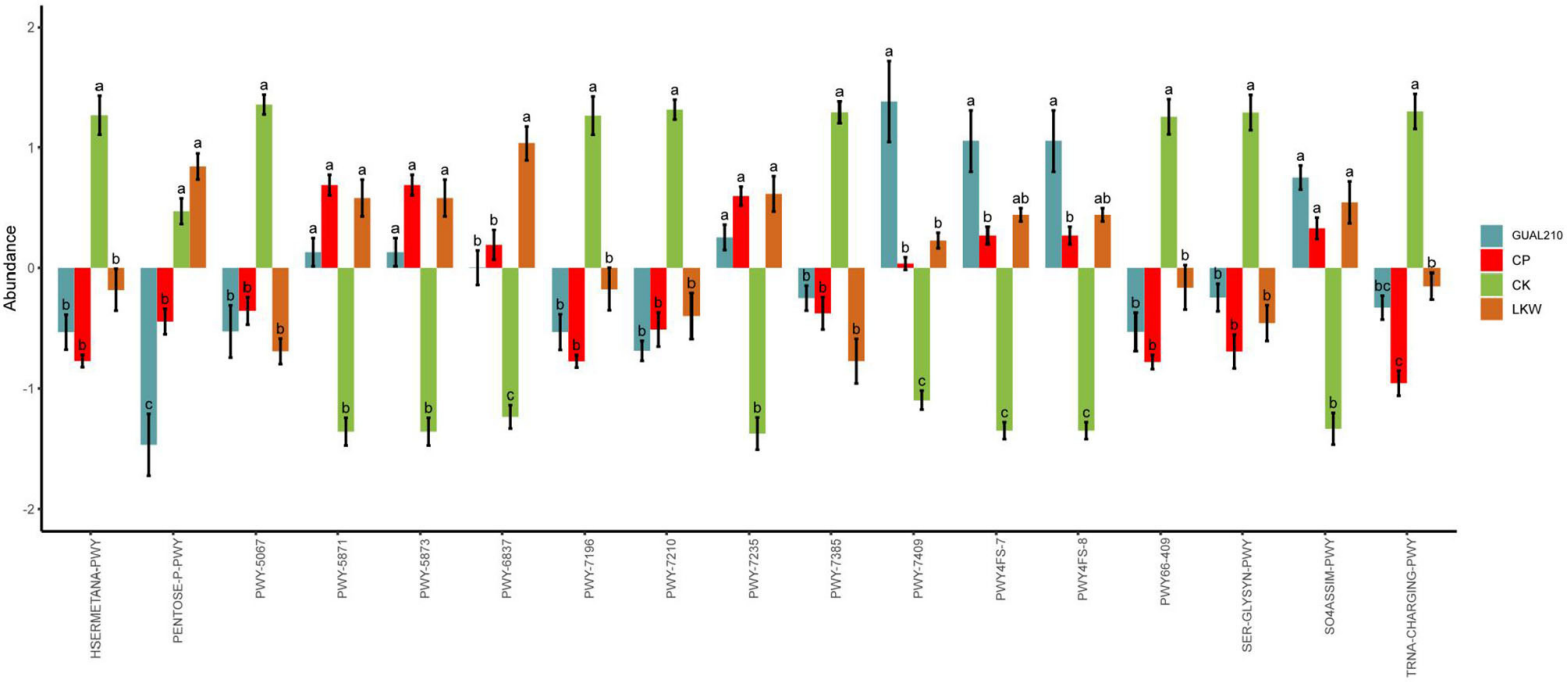
MetaCyc pathway with significant differences obtained by ANOVA and Duncan test. The abscissa is the path name; for each pathway, different colors represent different groups. If there are the same letters above the two groups, the difference is not significant, otherwise the difference is significant.

The analysis of metabolic pathways (Figure 10) showed that there were significant differences between each experimental group and control group in the methionine synthesis pathway (HSERMETANA-PWY), L-serine and glycine biosynthesis super pathway I (SER-GLYSYN-PWY), sulfate reduction I (assimilation) pathway (SO4ASSIM-PWY), and between GUAL210 and other treatment groups in the pentose phosphate pathway (PENTOSE-P-PWY) and phospholipid remodeling pathway (PWY-7409). Among them, GUAL210 and LKW also contributed to the effects of phosphatidylglycerol biosynthesis I and II pathways (PWY4FS-7 and PWY4FS-8). LKW had significantly contributed to fatty acid beta-oxidation V (PWY-6837). There were significant differences in tRNA charging (TRNA-CHARGING-PWY) among the groups.

## 4. Discussion

### 4.1. Effects of biological control on disease prevention and plant growth promotion

**Figure 11:**
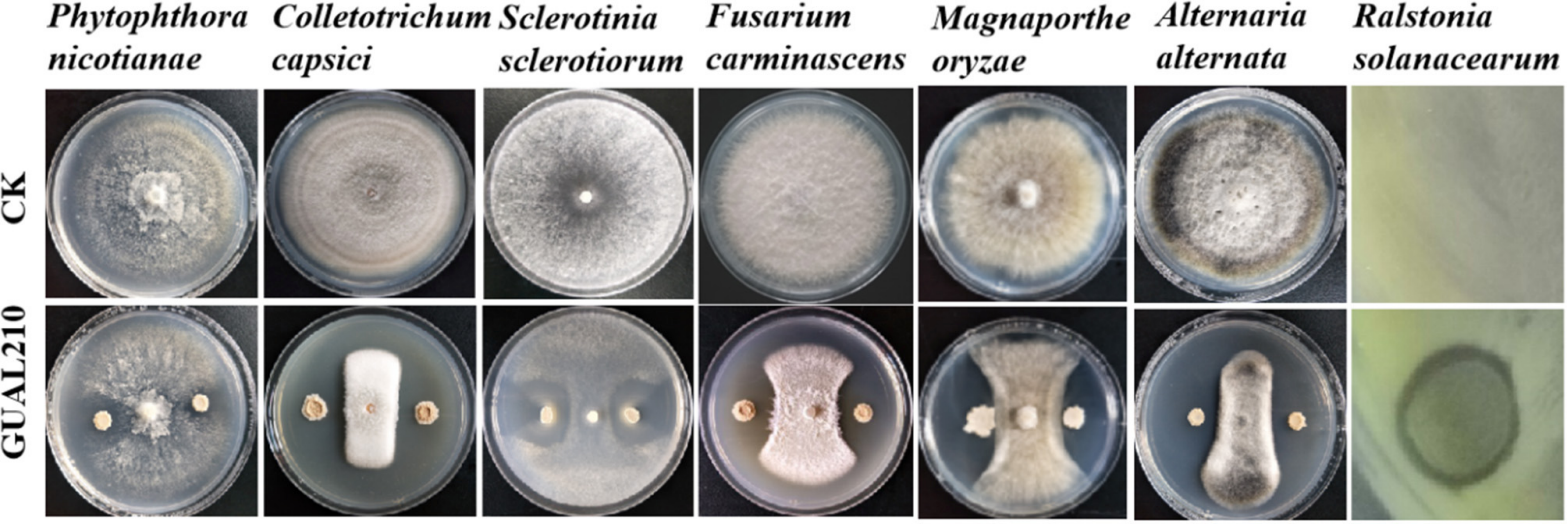
Antagonistic activity of *B. velezensis* GUAL210 against various plant pathogens in a dual culture test (Cheng, 2020; Zhou et al., 2023). (Antagonistic activity against *Colletotrichum capsici, Alternaria alternata, Phytophthora nicotianae, Sclerotinia sclerotiorum*, and *Magnaporthe oryzae* were reported by Cheng et al.).

The genus *Bacillus* includes many beneficial species, such as *B. subtilis, B. amyloliquefaciens, B. thuringiensis, B. lichgxeniformis*, and *Paenibacillus polymyxa*, which are widely used in plant disease control and research and development of biocontrol agents (Shafi et al., 2017). The genus *Bacillus* is one of the most important groups of plant growth-promoting rhizobacteria (PGPR) that can produce antifungal substances, such as subtilisin and surfactants, and facilitate the growth of rhizosphere and plant disease control (Yang, 2015). *B. velezensis* is an important member of the genus *Bacillus*, and *B. velezensis* GUAL210 can be isolated from the rhizosphere of healthy pepper plants growing in high-incidence anthracnose fields in Guizhou, China (Cheng et al., 2019). Previous research has shown that GUAL210 produces enzymes with biocontrol activity, such as proteases, cellulases, siderophores, and phosphatases, and forms a robust biofilm at the liquid–air interface when grown in liquid culture without agitation and displays a robust swarming phenotype (Zhou et al., 2023). Previous experiments have found that it has obvious antagonistic effects on many plant pathogenic fungi such as *Colletotrichum capsici* (pepper anthracnose), *Alternaria alternata* (sorghum leaf spot), *Phytophthora nicotianae* (tobacco black shank), *Phomopsis* sp. (pomegranate dry rot), *Sclerotinia sclerotiorum* (rape sclerotium), and *Magnaporthe oryzae* (rice blast) (Figure 11). In this study, the disease incidence and DI under the treatment of biological control were significantly lower than those under the treatment of chemical control. GUAL210 and LKW exhibited no significant difference in RCE and were superior to chemical control in terms of disease control effects and duration. In addition, biological control significantly affected the diameter growth and branch and flower number of rose plants, showing greater growth-promoting effects than chemical control. Correlation analysis showed that *Exophiala, Conlarium* (in GUAL210), *Hamigera, Entorrhiza, Clavulina*, and *Odontia* (in LKW) may facilitate the growth of roses, while *Minutisphaera, Thermomyces, Mortierella*, and *Neosetophoma* (in CK) may impede the growth of roses. However, further studies are needed to consolidate these findings.

### 4.2. Analysis of fungal community structure and species diversity

Black spot disease is mainly caused by *M. rosae* (Lib.) Fr. (Feng and Li, 2019). In this study, the smooth and hairless leaves of *R. chinensis* were likely to be a hotbed of pathogen growth (Dong et al., 2020). The pathogens of black spot disease can overwinter in the form of sclerotia, mycelia, and conidia in plant-diseased tissues in the soil, so soil environments and rhizosphere fungal communities have a close relationship with the incidence of rose black spot disease. Most pathogens belonged to the phylum Ascomycota. The relative abundance of Ascomycota was the highest in CK, followed by GUAL210, CP, and LKW, which was consistent with the disease occurrence in each group. The dominance of the phylum Basidiomycota was the highest in LKW, followed by GUAL210 and CP, which may be related to the health of the rose plant. Diversity analysis showed that both chemical and biological control reduced the diversity of fungal communities, among which LKW had the most significant effect. Similarly, the application of biological and chemical control agents has been reported to effectively reduce the rhizosphere fungal abundance of eggplant (Bozena and Nowak, 2011).

### 4.3. Pathway function prediction and analysis of fungal community

The analysis of metabolic pathways showed that the HSERMETANA-PWY in each experimental group was significantly lower than that in the control group, which was consistent with the actual disease status. The synthesis of methionine had a significant effect on the pathogenicity of fungi. The blockage of the HSERMETANA-PWY can lead to a decrease in the pathogenicity of various pathogenic fungi (Fu et al., 2013). Phosphatidylglycerol had a physiological role in participating in the construction of photosynthetic membranes and regulating various metabolic activities such as photosynthesis. The inhibition of synthesis can directly affect photosynthesis and thus affect plant growth (Chen et al., 2007). GUAL210 and LKW were significantly higher than CK in phosphatidylglycerol synthesis pathways PWY4FS-7 and PWY4FS-8. The results also showed that there were significant differences in L-serine and glycine synthesis metabolic pathways between each group. Fungi in the rhizosphere soil of roses may indirectly affect the growth and flowering of roses through metabolites and transformation of these substances.

### 4.4. Biological control optimized the dominant fungal flora

Through parasitism, nutrient competition, and resistance induction, the saprophytic fungus *Trichoderma* has a better control effect on plant diseases than chemical control agents (Chen and Wei, 2015; Khan et al., 2021). In addition, *Trichoderma* can also promote plant growth and enhance stress resistance (Pereira et al., 2020; Rodrigues et al., 2021). *Fusarium* is a widely distributed causal pathogen responsible for plant diseases such as branch blight, wilt and stem, and root rot (Singh et al., 2021; Chen et al., 2022; Gibert et al., 2022). The results showed that *Trichoderma* was the dominant flora in GUAL210, the abundance of *Fusarium* was the lowest in GUAL210, and the abundance of *Trichoderma* was relatively low in CP and CK, indicating that there may be a cozy symbiosis between *Trichoderma* and *B. velezensis* GUAL210. Similarly, it has been reported that *Trichoderma* and *B. amyloliquefaciens* have compatible growth characteristics (Yang, 2018). The relative abundance of *Paraphaeosphaeria* was the highest in GUAL210, and its various metabolites have antifungal activity (Amorim et al., 2019; Carrieri et al., 2020; Chen et al., 2022), so it may be disease-resistant flora. Under the treatment of broad-spectrum fungicides, *Penicillium* became the dominant flora. The most severe black spot disease was found in CK. It has been reported that *Alternaria* and *Mortierella* are the dominant rhizosphere flora of potato Verticillium wilt (Zhao et al., 2021) and tobacco black shank (Xiang et al., 2019). The relative abundance of *Mortierella, Scutellinia, Thermomyces*, and *Pestalotiopsis* was the highest in CK, while the relative abundance of *Mortierella* in GUAL210, LKW, and CP was significantly lower. *Mortierella* and *Chaetomiaceae* had the highest correlation with DI. *Pestalotiopsis, Alternaria*, and *Mortierella* may be the dominant flora that caused rose disease. *Pestalotiopsis* is known to be the causal pathogen of many plants. For instance, *P. clavispora* (Zhao et al., 2014; Feng et al., 2015) and *P. versicolor* cause gray leaf spot of *Camellia japonica* and wilt of *Myrica rubra* (Ren et al., 2013). The abundance of known pathogens, such as *Ustilaginoidea* and *Paraconiothyrium*, was higher in CP and CK but lower in GUAL210 and LKW, indicating that biological control had an inhibitory effect on pathogens, while chemical control failed to prevent the invasion of pathogens.

In summary, differential aggregations of characteristic fungi determined the health of the rhizosphere ecosystem and the disease incidence of rose plants. *Trichoderma, Paraphaeosphaeria, Suillus, and Umbelopsis* in GUAL210 and *Galerina* in LKW replaced *Mortierella, Pestalotiopsis, Ustilaginoidea, Paraconiothyrium, Fusarium*, and *Alternaria* as dominant flora and played a non-neglectable role in reducing plant disease occurrence.

## 5. Conclusion

The difference in rhizosphere fungal community structure had an important impact on the incidence of black spot disease. Biocontrol bacteria helped improve rhizosphere fungal community structure and maintain a healthy soil microbial ecosystem, which further contributed to the prevention of plant diseases. GUAL210 has promising prospects for application and development and maybe a good substitute for chemical control agents.

## Data availability statement

The sequencing data for this study have been deposited in the NCBI database under accession numbers SRR25296951–SRR25296968.

## Author contributions

WD is the experimental designer and executor of this study. JM and TL participated in data collation, data statistics, and the writing of the first draft of the manuscript. NW, JJ, WM, and ZZ participated in some experiments. TL assisted in the translation and polishing of the thesis. HZ and HD jointly guide the experimental design, thesis writing, and revision. All authors agreed on the final text. All authors contributed to the article and approved the submitted version.
